# Editorial: Comorbidity in patients with psychiatric disorders: Epidemiological and molecular perspectives

**DOI:** 10.3389/fpsyt.2022.1108922

**Published:** 2023-01-05

**Authors:** Yezhe Lin, Liliang Li, Fanglin Guan, Dou Yin

**Affiliations:** ^1^Department of Psychiatry and Behavioral Science, Virginia Tech Carilion School of Medicine, Carilion Clinic, Roanoke, VA, United States; ^2^Clinical Research Center for Mental Disorders, Chinese-German Institute of Mental Health, Shanghai Pudong New Area Mental Health Center, School of Medicine, Tongji University, Shanghai, China; ^3^Department of Forensic Medicine, School of Basic Medical Sciences, Fudan University, Shanghai, China; ^4^Xi'an Jiaotong University Health Science Center, Xi'an, Shaanxi, China; ^5^Ruijin Hospital, School of Medicine, Shanghai Jiao Tong University, Shanghai, China

**Keywords:** multidisciplinary, comorbidity, psychiatric disorder, epidemiology, molecular, forensic

## Introduction

While psychiatric disorders are becoming the leading causes of global burdens in recent years (1), their comorbidities are prevalent and invariably affect individual's quality of life and life expectancy (2, 3). The disparities and discrepancies of comorbidities in different mental disorders indicate the potential shared underlying mechanisms. Comorbidity was recognized as one of the major contributors to unnatural deaths (e.g., suicide, homicide, or accident etc.) and natural deaths (e.g., sudden cardiac deaths). Given the complexity of the etiologies and interventions for comorbidities of psychiatric disorders (4), studies usually require a multidisciplinary approach, from public health to microscope, from epidemiology to molecular biology.

## Epidemiology

In a secondary analysis from the 2016–2018 National Inpatient Sample Dataset (NISD) on adolescents aged 12–17, Sun et al. investigated the prevalence and comorbidities of homicidal ideation (HI) in this young population. They found that in a total of 18,935 patients (mean age: 14.5) with the diagnosis of HI, patients were more likely to be male compared to the control group (58.7 vs. 41.2%, *p* < 0.001). Major depression [Odds ratio (OR): 2.66, *p* < 0.001], bipolar disorder (OR: 3.52, *p* < 0.001), anxiety disorder (OR: 1.85, *p* < 0.001), ADHD and other conduct disorders (OR: 4.01, *p* < 0.001), schizophrenia (OR: 4.35, *p* < 0.001) are significantly more common in adolescents with HI. Furthermore, suicidality was prevalent in 66.9% of patients with HI.

## Mechanisms of comorbidity and interventions

Chen, Ju, et al. and Zhu et al. (5) explored the psychological and molecular mechanisms underlying the highly overlapping condition of depression and pain. Catastrophizing and anxious thoughts could mediate comorbid depression and chronic pain (Chen, Ju, et al.). In the mice model of comorbid depression and chronic pain developed by the administration of dextran sodium sulfate (DSS) and the induction of chronic unpredictable psychological stress (CUS), the authors found that both mRNA and protein levels for Iba-1, Brain-Derived Neurotrophic Factor (BDNF), and cAMP response element-binding protein (CREB) were decreased in line with the lower microglia density in the medial prefrontal cortex (mPFC) (5).

Metabolic side effects induced by second-generation antipsychotics (SGA) remain a serious issue and could draw back their therapeutic effect (6). It was indicated in adult studies that metformin could counteract the metabolic side effects of SGA (7). A meta-analysis by Mansuri et al. collectively explored the therapeutic and side effects of metformin in children and adolescents treated with SGA from randomized controlled trials. In a total of 213 patients (metformin: 106; control:107), 12–16 weeks of metformin therapy had a significant reduction in weight [mean difference (MD): −4.53 lbs, *p*-value < 0.001], BMI *z* score (MD, −0.09, *p*-value: 0.004), and insulin resistance (MD: −1.38, *p*-value: 0.002) compared to controls. However, more nausea-vomiting (OR: 4.07, *p*-value: 0.02), and diarrhea (OR: 2.93, *p*-value: 0.002) were observed in the metformin group.

## Addictive disorders—A genetic level of multimodal perspectives

Substance use disorder (SUD) or addictive disorders is a cluster of comorbidities among psychiatric and medical conditions evidenced by multiple studies. SUD could be a risk factor for other disorders or contributed by them. For example, SUD was observed in around half of the individuals with diagnoses of schizophrenia or bipolar disorder (8). Heavy substance uses also led to various health conditions and outcomes (9). An increasing body of research explored the genetic predisposition of SUD (8).

Xiao et al. screened 14 single nucleotide polymorphisms (SNPs) located within ±3 kb regions of histone deacetylase 3 (HDAC3) among 1,221 patients with or without methamphetamine dependence and found the A allele (minor allele) of rs14251 located at 5q31.3 associated with an increased risk of methamphetamine dependence in Han Chinese population. Though the SNP rs14251 could potentially affected quantitative expression signal for FCH And Double SH3 Domains 1 (FCHSD1), Protocadherin Gamma Subfamily B, 6 (PCDHGB6), and RELT Like 2 (RELL2) yet not for HDAC3, in various human tissues *in silico* analyses. Li B. et al. applied RNA sequencing (RNA-seq) to examine the expression profiles of Long Non-coding RNA (LncRNA), MicroRNA (miRNA), and mRNAs on the nucleus accumbens (NAc) of morphine addiction mice model built with morphine or saline conditioned place preference (CPP). Their study unveiled the potential Lnc15qD3-miR-139-3p-Lrp2 ceRNA interaction and shed some light into the network of RNA regulatory systems for opioid addiction.

## Forensic studies discover more dimensions in the comorbidity study

Wang et al. uncovered a research hotspot on the mechanisms of cardiotoxicity, the safety monitoring, and the assessment of the risk-benefit during clinical use of some newer antipsychotics, clozapine and olanzapine Web of Science core collections to inform the future research directions of antipsychotics-Induced Sudden Cardiac Death (SCD).

Chen, Zhang, et al. present nine previous schizophrenia inpatients of sudden death during hospitalization. Seven cases (77.8%) died of organic heart diseases such as severe coronary artery atherosclerosis (*n* = 4), myocarditis (*n* = 1), cardiomyopathy (*n* = 1), and pulmonary thromboembolism (*n* = 1). Two cases remained unexplained after systemic autopsy and toxicological examinations. In an autopsy study, Wang et al. (10) revealed that insufficient antipsychotic treatment for patients with schizophrenia could lead to higher rate of sudden unexplained death in a Han Chinese population in Mainland China. Li X. et al. juxtaposed the relationship between personality characteristics and craniocerebral trauma from a traffic accident patient population in Shanghai, China. In the exam of the simplified Neuroticism Extraversion Openness Five-Factor Inventory (NEO-FFI), craniocerebral trauma might lead to higher neuroticism, extraversion, and agreeableness scores, and lower conscientiousness scores in patients with personality disorder in a severity-dependent manner.

Our Research Topic “*Comorbidity in patients with psychiatric disorders: Epidemiological and molecular perspectives*” outlined a rough domain structure to make joined efforts to a deeper understanding of comorbidities of psychiatric disorders. Embracing a collaborative and multidisciplinary approach to study comorbidity could hopefully lead to the milestones on the mechanism and management of them in the future.

## Author contributions

YL and LL developed the ideas. YL drafted and finalized the manuscript. LL, FG, and DY participated in the revision. All authors contributed to the article and approved the submitted version.
